# Virtual multi-alignment theory of parallel-beam CT image reconstruction for rigid objects

**DOI:** 10.1038/s41598-019-49995-0

**Published:** 2019-09-18

**Authors:** Kyungtaek Jun

**Affiliations:** IM Technology Research Center, 6, Teheran-ro 52-gil, Gangnam-gu, Seoul, 06211 Korea

**Keywords:** Applied mathematics, Computational science

## Abstract

X-ray computed tomography has become an essential technique in various fields, such as biology, archaeology, geoscience, and materials science. Although considerable effort has been given to reconstructing projection image sets from fixed rigid specimens, little attention has been given to the reconstruction of projected images using an alignment method with a rotation axis for objects that are moving during scanning. Therefore, in this study, a mathematical solution for the reconstruction of a projection image set that is obtained from moving objects is presented. In particular, this study presents the reconstruction of a projection image set for two and three objects moving in different directions using the virtual multi-alignment method.

## Introduction

X-ray computed tomography (CT) has been studied due to its potential applications in various fields such as biology, archaeology, geosciences, and materials science^1–6^. CT is a nondestructive imaging technique for visualizing the internal structures within rigid objects and obtaining digital information about shapes and properties in a three-dimensional (3D) space based on two-dimensional (2D) projections. This goal is achieved by penetrating objects with X-rays at different angles.

In the field of computer vision, many researchers have proposed methods to eliminate translation and tilt errors in aligned reconstructions; in particular, many studies related to alignment algorithms and image matching have been performed to address translation and tilt error problems^2,7–11^ by finding the rotation axis using a rearranged projection image set^12^. Recently, Jun and Yoon proposed a new alignment solution to reconstruct CT images using fixed points and virtual rotation axes^7^; the proposed method restored images and provided promising results in terms of eliminating the translation errors of the rotation axis and specimen and the vertical tilt errors of the rotation axis, which is the tilt of the rotation axis in the direction that is orthogonal to the X-ray beam, using a virtual rotation axis. Furthermore, Jun and Kim proposed an alignment theory for reconstructing the images of elastic objects undergoing specific motions using the virtual alignment method (VAM), which was called the virtual focusing method^7,13^.

In particular, to obtain an ideally aligned reconstruction (IAR) that does not include translation and tilt errors, the projection set that is obtained during scanning should be well aligned to satisfy the Helgason-Ludwig consistency condition for a projection angle of up to 180°; the projection set should contain all the information about the specimen or desired region for which the reconstructed image is to be obtained. In a rigid object, if there is movement in every specimen during the process of obtaining a projection set, mathematical modifications are required to obtain an aligned reconstruction because the movement of specific regions within the specimen during scanning causes translation errors. In this study, we investigate the motion that occurs when rigid objects move during scanning. In particular, we present mathematical approaches for the reconstruction of a rigid specimen in which the motion of the objects within the specimen is captured using the VAM. Furthermore, we present a solution for the creation of a sinogram for each rigid section within a rigid specimen by creating a pair of ideal sinogram patterns (ISPs) for each section of the sample without the need for an overall ISP, and we refer to this approach as the virtual multi-alignment method (VMAM). The primary advantage of this method is that an IAR can be obtained by aligning the desired part of an object in the sinogram when an object moves in various directions during scanning. Based on the numerical results, this technique showed promising performance in terms of reconstructing the images that are obtained from moving rigid objects.

## Fundamental Necessity of the Virtual Multi-Alignment Method

A sinogram that is obtained without any translation or tilt errors from a rigid sample during scanning constitutes an ISP. Therefore, every point of an object in a specimen moves along a continuous sinusoidal function in the sinogram while rotating about its rotation axis. Such a sinogram can be used to obtain an IAR. If the sinusoidal function representing the orbit of a point in an object has a discontinuity at a certain projection angle or if there are translation and/or tilt errors while obtaining the projection set, the sinogram constitutes a non-ideal sinogram pattern (NISP). It is difficult to obtain an IAR using an NISP because of the errors. Thus, to obtain an IAR, an NISP should be transformed into an ISP using mathematical modifications.

**Figure 1:**
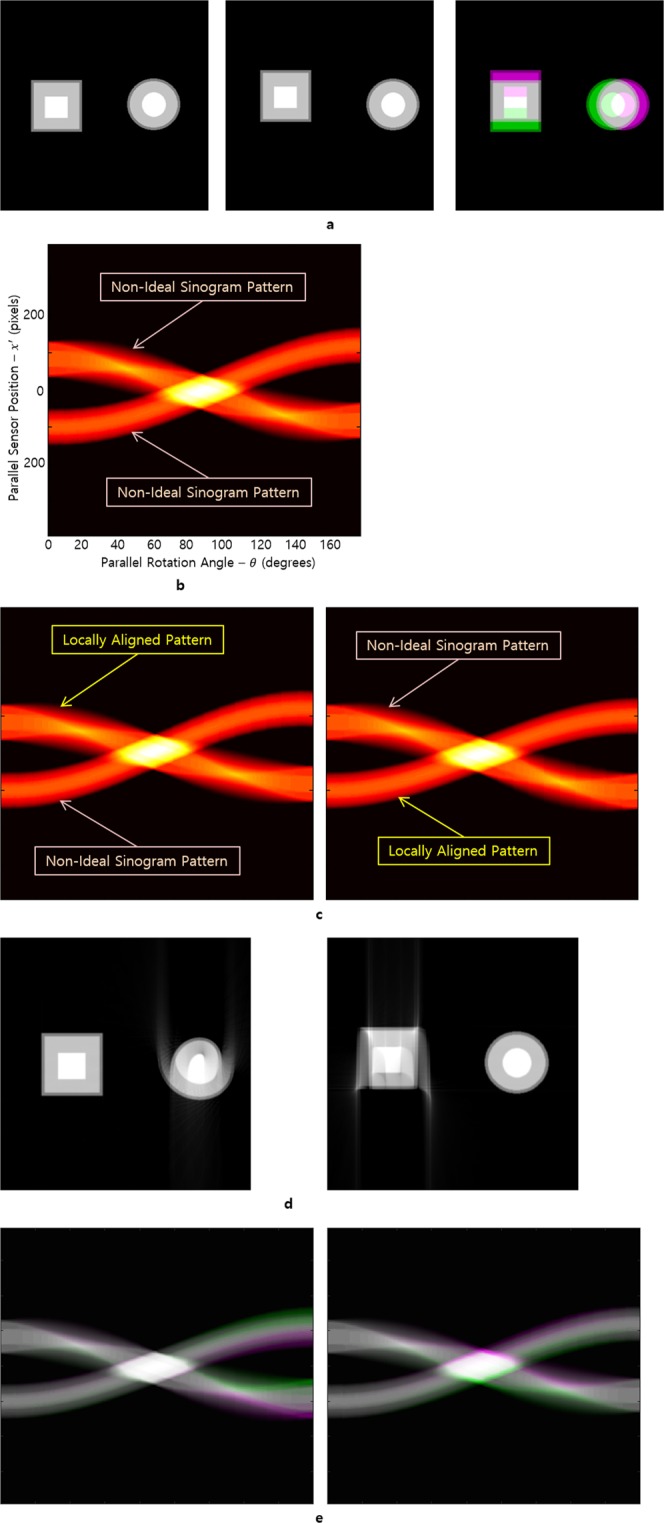
Reconstruction procedure for two rigid bodies with movement during scanning. In particular, the square moves up and the circle moves right. The two objects have been moved by one pixel per projection angle of 9°. (**a**) The structure of the image specimen was captured at different projection angles (0°, 89.85°, and 179.85°, which correspond to left, middle, and right, respectively). (**b**) The overall sinogram for the two objects moving slowly and continuously during scanning. (**c**) The position of each pixel or point of the rectangle is adjusted to satisfy the sinusoidal function ${T}_{{r}_{n},{\phi }_{n}}(\theta )\,\,$for each initial position to construct the LAP for the rectangle (left). A similar method is used to construct an LAP for the circle (right). (**d**) Locally aligned reconstructions that are obtained using the LAP of each sinogram in (**c**). (**e**) The left image shows the difference between the sinograms shown in (**b**) and (c-left). The density of each white pixel in the sinogram in (e-left) is the same as in picture (**b**) and (c-left). The density of the purple pixels in (e-left) corresponds to higher values in the sinogram of (**b**) than values of the sinogram used for (c-left). On the contrary, the density of the green pixels in (e-left) corresponds to higher values in the sinogram of (c-left) than the one in (**b**). The right image in (**e**) shows the difference between the sinogram shown in (**b**) and (c-right).

Figure 1 depicts the reconstruction process for the case when errors occur when two rigid objects (a square and a circle) move in standard directions (i.e., the x- and y-directions). The positions of two pairs of objects in an image sample are captured at the projection angles *θ* = 0° (Fig. 1a, left) and *θ* = 179.85° (Fig. 1a, middle). In this study, the two rigid objects moved in different directions during scanning. Object A (square) moved up, while object B (circle) moved to the right by one pixel per projection angle of 9° (Fig. 1a, right). The overall sinogram for the objects that are moving slowly and continuously is shown in Fig. 1b. For each projection angle in the sinogram, an object translation error occurs, which appears as an NISP in the sinogram. Although it is possible to predict and trace the trajectory of objects A and B using the sinogram, it is not easy to correctly classify each of them and correct the errors in the projection images. Thus, to transform the NISPs to the ISP, it is necessary to create an ISP corresponding only to object A to obtain a reconstructed image of object A, and similarly, an ISP corresponding only to object B to obtain a reconstructed image of object B (see the Methods section). When translation errors occur as an object move during scanning, IAR can be performed by correcting the sinogram of the previous entire part or following entire part of the error in the ISP using the VAM. In addition, this method can be applied to two rigid objects within a specimen. Thus, it is possible to convert an NISP describing the motion of a desired rigid object that includes translation errors to a locally aligned pattern (LAP) using the VAM (Fig. 1c). The LAP is obtained by converting the NISP of each section to the ISP using the mathematical alignment method (VAM with fixed points). Furthermore, we used an algorithm to modify the vertical translation and parallel translation errors to obtain the LAP (Fig. 1c left) of object A and the LAP (Fig. 1c right) of object B. The sinogram with the LAP can then be used to reconstruct the sample image before the movement occurred at the desired projection angle using the region of interest (Fig. 1d).

**Figure 2:**
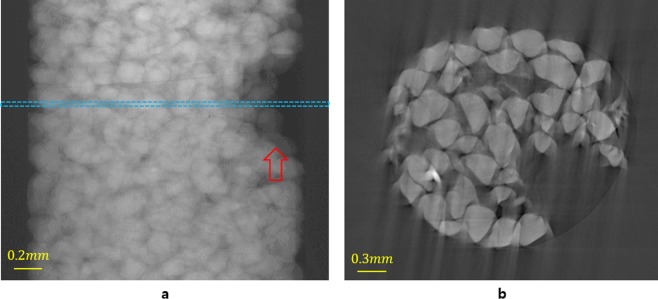
A specimen in which the soil grains move during scanning. The figure represents the dissolution of the grains, which is used as experimental data. The projection image and reconstructed slice of Hanford soil in a PEEK column are obtained using the National Synchrotron Light Source X2B beamline at the BNL. A clean reconstructed image cannot be created because large holes are present in the actual sample, which cause small movements of the grains inside the PEEK during the scanning of the set of 1200 projection images. (**a**) The projection image of column “S1” with a large hole that changed during scanning by the movement of soil grains because of dissolution and other effects. The red arrow indicates a large hole, and the dashed line indicates the axial level that is used for the reconstruction in (**b**). (**b**) The reconstructed slice using an NISP.

Figure 2 shows a reconstruction with an NISP and the experimentally obtained projection image from the experiment involving the dissolution of the grains in a sample. This experiment was conducted at the Brookhaven National Laboratory (BNL), the Pacific Northwest National Laboratory (PNNL), Stony Brook University, and Princeton University to observe the secondary mineral precipitates that are caused by Al and the dissolution on the quartz surface without Al. Initially, the soil grains were fully filled in the polyether ether ketone (PEEK). Although there are multiple large holes inside the PEEK, they are situated in a stable conformation. However, during the experiment, due to the dissolution of the grains, the soil grains moved and the large hole changed its shape (Fig. 2a). This projection image set consists of 1200 projection images that were obtained at the microtomography beamline with a 15 keV for approximately 4 hours. Since the energy of the beamline affects the specimen, the incident intensity of the specimen, or the flat field image, is measured every 60 projection images. The size of the projection image is 1340 × 500 pixels and the pixel size is 4 *μm*. During this process, the soil grains are affected by the formed holes and small random movements of these grains tend to occur. In this case, there is no ISP in the sinogram; therefore, a clean image cannot be obtained (Fig. 2b). To obtain an IAR without the errors that are mentioned above, we provide a mathematical solution that transforms an NISP into each LAP using two or three rigid objects that are moving in different directions.

## Rigid Multi-Body System

**Figure 3:**
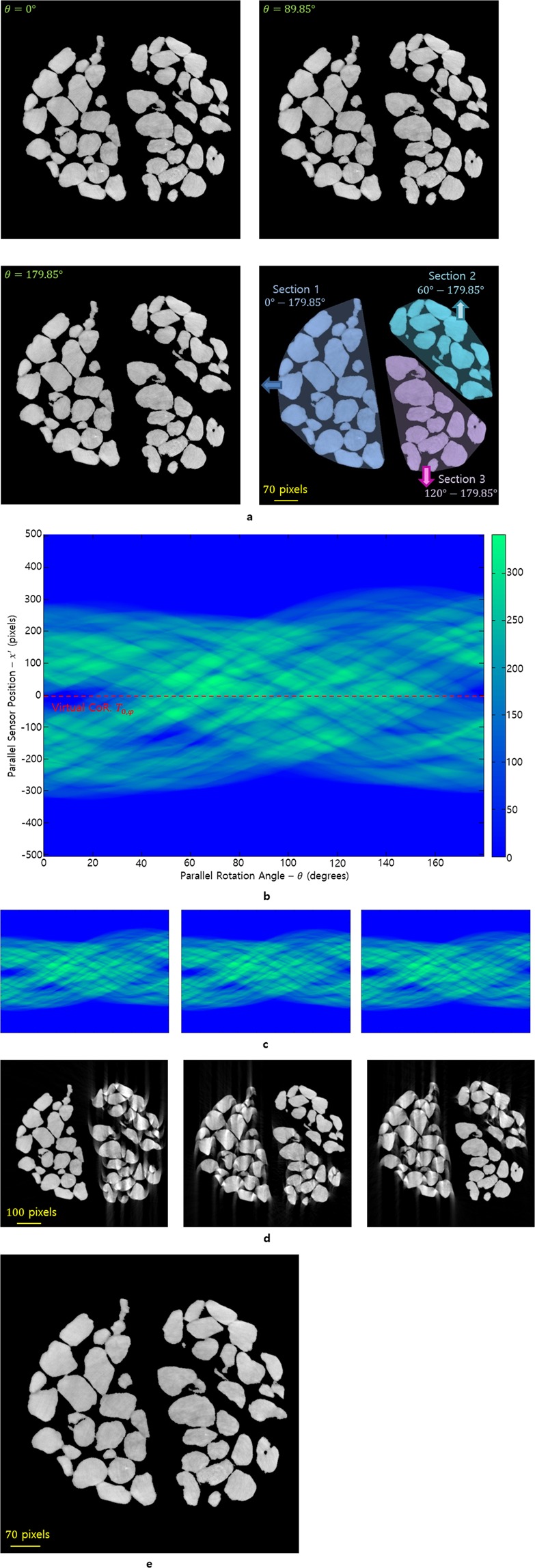
Reconstruction process explanation when movement errors occur in three different rigid sections (Section 1, 2, and 3) within a sample. The image sample is 714 × 714 pixels and has a pixel width of 4 *μm*. (**a**) The structure of the image specimen was captured at different projection angles (0°, 89.85°, and 179.85°, which correspond to top left, right and bottom left, respectively). Each section moves in the direction that is indicated the arrow by one pixel per projection angle of 9° (bottom right). (**b**) The overall sinogram for the three sections moving slowly and continuously during scanning. The red line represents the virtual rotation axis ${T}_{0,\phi }(\theta )\,\,$for the VAM. (**c**) These figures are the sinograms that are obtained by transforming the NISP of each section using the sinogram in (**b**) into LAPs using the VMAM. In particular, the positions of each pixel or point of each section is adjusted to satisfy the sinusoidal function ${T}_{{r}_{n},{\phi }_{n}}(\theta )\,\,$for each initial position to obtain the LAP for Sections 1 (left), 2 (middle), and 3 (right). (**d**) The locally aligned reconstruction using each sinogram that was obtained from (**c**). (**e**) Ideally multi-aligned reconstruction combing the locally aligned reconstructed sections from (**d**) through each section’s convex hull (region of interest).

To illustrate the accuracy of the reconstruction image obtained by the VMAM, we used a reconstructed slice with weak ring artifacts as a test image sample. When three or more rigid objects move within a specimen, the VMAM can also be applied to convert the NISPs describing the object trajectories in the sinogram into LAPs. In addition, the VMAM can be applied even when the rigid objects are moved at different times. If information is available on the manner in which each rigid object moved from one projection angle to another, reconstruction is possible using the VAM for each moved section. Figure 3 shows a solid grain image that is scanned at the BNL that consists of three rigid sections (labeled Sections 1, 2, and 3). Figure 3a shows the structure of the image sample at each angle of 0°, 89.85°, and 179.85° (which correspond to top left, top right and bottom left, respectively). In particular, Section 1 moves to left from 0° to 179.85°, Section 2 moves up from 60° to 179.85°, and Section 3 moves down from 120° to 179.85° during scanning. In the sample images, a pixel corresponding to a void has a value of 0, whereas one corresponding to a grain particle has a specific nonzero value. In addition, the image specimen has weak ring artifacts in the three sections; furthermore, each section consists of pores and grains. Therefore, it is necessary to evaluate whether these microstructures are precisely reconstructed after movements occur. It is very important to correct the translation errors that occur as the three sections move and convert the NISP to the LAP for each section because the sinogram is modified by dividing the specimen into those before and after movements. After obtaining the sinogram describing the overall movements during scanning (Fig. 3b), the LAP (Fig. 3c) can be reconstructed using the filtered inverse Radon transform (Fig. 3d). In particular, the sample image can be reconstructed to the position of the object that is desired using the region of interest (Fig. 3e).

## Results and Discussion

The numerical results show that when two or more rigid objects move within a sample during scanning, the image for the desired region can be obtained using the VMAM. Furthermore, even if a specimen consists of more than two sections, it is still possible to reconstruct images. While using the VMAM, it is not important to know the number of movements that occurred or the number of sections within a specimen that moved during scanning; instead, it is necessary to classify and modify the projection images or sinogram before and after the movement.

**Figure 4:**
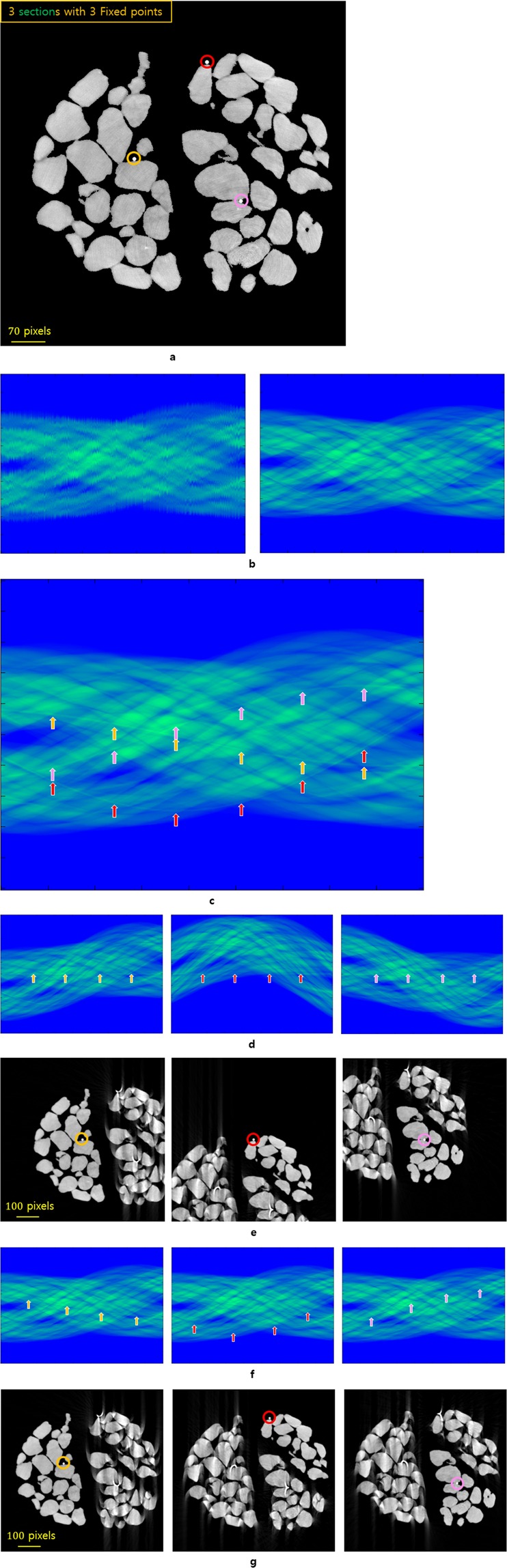
Ideally multi-aligned reconstruction for the moving rigid sections within a specimen using the VMAM through fixed points. The image sample is 714 × 714 pixels, and the pixel width is 4 *μm*. (**a**) The image sample at the projection angle of 0°. Each section contains one fixed point (the yellow, red, and pink circles represent the fixed points for Sections 1, 2, and 3, respectively). (**b**) The sinogram with vibration that was obtained using the high-resolution beam-type (left). A rearranged sinogram using the center of attenuation or center of mass (right). (**c**) The overall sinogram for the three sections that are moving slowly and continuously during scanning. The different colored arrows indicate the trajectory of each fixed point. (**d**) The sinograms that are obtained by moving the fixed point of each section along the orbit of the virtual rotation axis ${T}_{0,\phi }(\theta )$ such that the density of all pixels in each section satisfies ${T}_{{r}_{n},{\phi }_{n}}(\theta )$. (**e**) The locally aligned reconstructions using the sinograms that were obtained in (**d**). (**f**) The sinograms that are obtained by moving the centers of the fixed points of each section along its initial sinusoidal function ${T}_{r,\phi }(\theta )$ such that the density of all pixels in each section satisfies ${T}_{{r}_{n},{\phi }_{n}}(\theta )$. Each sinogram has an LAP. In addition, the VAM is used to eliminate the parallel and vertical translation errors that occur as each section moves. (**g**) The locally aligned reconstructions using each sinogram that was obtained from (**f**).

If there are more than two moving patterns of objects within a specimen consisting of several rigid objects, it is not possible to create an ISP for the reconstruction for the overall motion of the object. In this case, an LAP should be created for each object. To obtain the IAR of each object, the motion of the sample should either be calculated, or a sufficient number of fixed points is required (Fig. 4). To determine whether the sinogram of the section containing the fixed point is an LAP, it is sufficient to evaluate whether the trajectory of the fixed point in the sinogram satisfies the sinusoidal function ${T}_{r,\phi }(\theta )$ (see the Methods section). If the fixed points of objects move along the circular trajectory in real space, every single point in the rigid section satisfies the sinusoidal function ${T}_{{r}_{n},{\phi }_{n}}(\theta )$ in the sinogram and the resulting sinogram has the LAP. The necessary condition for creating an IAR is that alignment is required until all fixed points satisfy the function, ${T}_{{r}_{n},{\phi }_{n}}(\theta )$.

**Figure 5:**
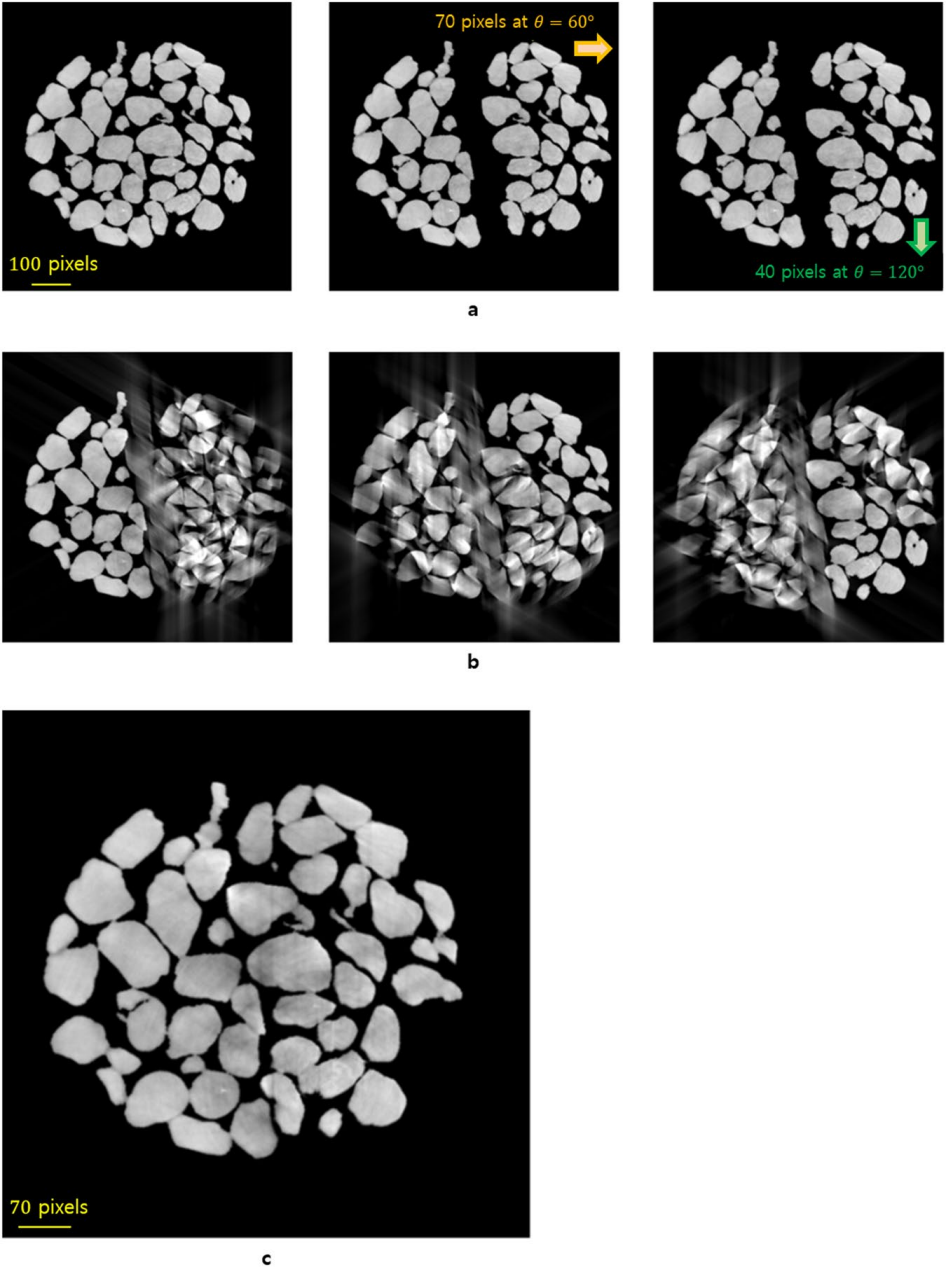
The effects of interference on the reconstructions when objects that are close to each other suddenly make large movements during scanning. (**a**) The structure of the image specimen was captured at different projection angles (0°, 89.85°, and 179.85°, which correspond to left, middle, and right, respectively). The right two sections of the sample move right by 70 pixels at the projection angle θ = 60°, and then the bottom section also moves down by 40 pixels at the projection angle θ = 120°. (**b**) The reconstructed images obtained after application of the VAM for each section. (**c**) The final reconstructed image using the region of interest.

If rigid objects suddenly have large movements when they are adjacent to each other (Fig. 5), or if the density variation in the projection image set is large, the movement of other misaligned objects may affect the reconstruction even if an IAR from a locally ideal sinogram for one object is obtained. However, if there are rigid objects moving in different directions within a sample, the VMAM is essential to obtain a good aligned reconstruction. While using the VMAM, it is more important to find the number of fixed points throughout the projected image set; these fixed points also include the points that can be distinguished or calculated from projection images, such as piercing points^14^, fiducial markers^15–20^, centers of attenuation^7^, and centers of high- or low-density spots^7^. In this study, we present a general solution for a specimen consisting of several complex rigid objects.

## Methods

Consider a sample consisting of several soil grains. To observe the motion of these grains and find the fixed points in the images, it is necessary to create a rearranged projection image set by aligning the center of attenuation or center of mass^10,14,21^ of the specimen. Using the fixed points that are obtained from the rearranged projection image, the process of locally aligned reconstruction is repeated by finding a common layer^7^ for each grain, creating a sinogram, and converting it to an ISP. For convenience, we would like to describe a specimen without any motion in the axial level with each grain’s fixed point existing at one axial level.

### Virtual alignment method using a fixed point

The circular trajectory of a point p in real space corresponds to a curve that is drawn by the sinusoidal function in the sinogram. The sinusoidal function is given by the following:

1 $${T}_{r,\phi }(\theta )=r\,\ast \,\cos (\theta -\phi ),0\le \theta  < 180^\circ $$

where *r* is the distance between the rotation axis and point *p*, *θ* is the projection angle, and *φ* is the angle between the line $\overleftrightarrow{Op}$ and the orthogonal line to the projection angle for *θ* = 0.

In ideal cases, the center *O* is converted to ${T}_{0,\phi }(\theta )$ in the sinogram; however, this is not always true in an actual sinogram. In particular, ${T}_{r,\phi }(\theta )$ is a function that determines the manner in which a specific point *p* in real space moves on the sinogram. Therefore, if a point *p* in the solid specimen rotates and the projected curve that is drawn by the movement of *p* for each angle is the same as the sinusoidal curve that is obtained by ${T}_{r,\phi }(\theta )$ in the sinogram, then the projected trajectories of the other points in the specimen should satisfy the projected curves by ${T}_{{r}_{n},{\phi }_{n}}(\theta )$ as well.

### Ideally multi-aligned reconstruction for rigid sections

Consider a sample consisting of *n* rigid sections. In addition, assume that there is at least one fixed point in each section. To use the initial positions of sections before movements, we need to calculate the trajectory wherein each fixed point moves around the rotation axis using the projection images that are obtained at the projection angles before the translation errors occur during the image acquisition process. For each area after the translation error occurs, it is necessary to find the LAP by aligning each fixed point to the trajectory of the fixed point before the error occurs. Then, all the projected sections in the sinogram represent the LAP. This indicates that every single point in the section moves along its own circular trajectory, which can be expressed as a sinusoidal function in the sinogram. However, it is difficult to find the rotation axis of these points because it requires considerable effort and time. In a previous work^7^, we changed the position of the reconstructed section to the desired position using the VAM via the fixed points and virtual rotation axis. However, in this work, we introduce an efficient method of IAR by applying the fixed point of each section to the virtual center of rotation ${T}_{0,\phi }(\theta )$ using the VAM. Here, we describe the proposed method using an image sample consisting of three rigid sections with each section containing a distinguishable high-density area (considered as a fixed point) (Fig. 4). As previously mentioned, to construct a sinogram with a locally aligned pattern for each section in a specimen composed of several rigid sections, there must be the same number of fixed points and rigid sections (Fig. 4a). To observe the trajectory of each fixed point in a sinogram, it is necessary to align the projection images on the axial level to the sinogram using the center of attenuation or center of mass (Fig. 4b). Then, we obtain the fixed points for each projection angle using the aligned sinogram (Fig. 4c). By applying each obtained fixed point to the trajectory of the virtual center of rotation ${T}_{0,\phi }(\theta )$ in the sinogram, we can produce a sinogram with a locally aligned pattern (Fig. 4d). We can construct a locally aligned reconstruction for each rigid section (Fig. 4e) using these sinograms (Fig. 4d).

**Figure 6:**
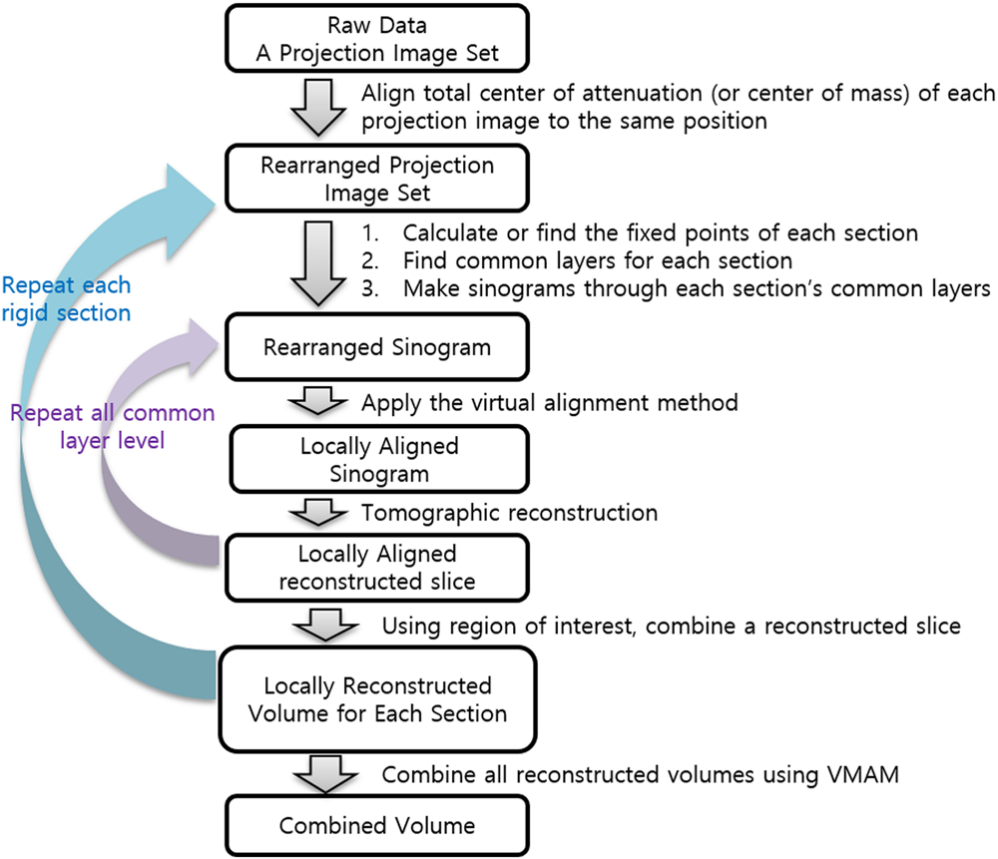
Schematic overview of the process for obtaining the 3D reconstructed volume using the VMAM. The trajectory of a fixed point can be determined using the rearranged projection image set even if the error is not corrected. To create a clean image for one rigid section, it is necessary to find at least one fixed point of the section. The common layer can be found using the trajectory of fixed points in the sinogram. Then, we can create a sinogram corresponding to each common layer and use the VAM to create an IAR of the section. By repeating these abovementioned steps for each rigid section, we obtain an aligned reconstructed volume for each section. The reconstructions for each section that are obtained using this method can be integrated using the region of interest to obtain the overall reconstructed volume.

A schematic overview of the process that is used for obtaining a 3D reconstructed volume using the VMAM is shown in Fig. 6. The proposed process for the ideally multi-aligned reconstruction in the common layer is described as follows.

**Step 1**: In the sinogram, rearrange the projection image by applying the center of attenuation or center of mass to the trajectory of ${T}_{0,\phi }(\theta )$ (Fig. 4b).

**Step 2**: Find or calculate the fixed point that corresponds to a part of each rigid section in the sinogram (Fig. 4c).

**Step 3**: Create a sinogram with a locally aligned pattern by placing one fixed point at the virtual center of rotation ${T}_{0,\phi }(\theta )$ (Fig. 4d).

**Step 4**: Use the obtained sinogram (Fig. 4d), and construct a locally aligned reconstruction (Fig. 4e).

**Step 5**: Repeat Steps 3 and 4 for each rigid section using each fixed point.

**Step 6**: Reconstruct the combined image using the region of interest of each section.

Using this method, the 3D reconstructed volume can be obtained from the section. Figure 4f,g show the results that are obtained when using the initial position of the actual rotation axis that is obtained before the movements of the sections occur and aligning the rest of the sinogram with ${T}_{r,\phi }(\theta )$. In general, however, it is not easy to determine the actual center of rotation from a sinogram. Nevertheless, using this method, when an error occurs as the section moves, there is no need to determine the actual center of rotation.

The virtual alignment method is required to use the virtual multi-alignment method. The algorithm and test codes used for the image sample, as well as the projection movies of the actual samples obtained by VAM, will be implemented and included in the TomoPy Package^9^. Also, the source codes and examples associated with this paper will be updated sequentially.
